# Adoption of antithrombotic stewardship and utilization of clinical decision support systems—A questionnaire-based survey in Dutch hospitals

**DOI:** 10.1371/journal.pone.0306033

**Published:** 2024-06-21

**Authors:** Jetske Graafsma, Joanna E. Klopotowska, Hieronymus J. Derijks, Ewoudt M. W. van de Garde, Rien H. L. Hoge, Marieke J. H. A. Kruip, Karina Meijer, Fatma Karapinar-Carkit, Patricia M. L. A. van den Bemt

**Affiliations:** 1 Department of Clinical Pharmacy and Pharmacology, University Medical Center Groningen, University of Groningen, Groningen, the Netherlands; 2 Department of Medical Informatics Amsterdam UMC, University of Amsterdam, Amsterdam, the Netherlands; 3 Amsterdam Public Health Institute, Amsterdam, the Netherlands; 4 Department of Pharmacy, Jeroen Bosch Hospital, Den Bosch, the Netherlands; 5 Department of Pharmacy, St. Antonius Hospital, Nieuwegein, Utrecht, the Netherlands; 6 Division Pharmacoepidemiology and Clinical Pharmacology, Utrecht University, Utrecht, the Netherlands; 7 Department of Pharmacy, Wilhelmina Hospital, Assen, the Netherlands; 8 Gaston Medical, Eindhoven, the Netherlands; 9 Department of Hematology, Erasmus MC, Erasmus University medical center, Rotterdam, the Netherlands; 10 Department of Hematology, University Medical Center Groningen, University of Groningen, Groningen, the Netherlands; 11 Department of Clinical Pharmacy & Toxicology, Maastricht University Medical Center+, Maastricht, the Netherlands; 12 CARIM School for Cardiovascular Diseases, Maastricht University, Maastricht, the Netherlands; Leiden University Medical Center, NETHERLANDS

## Abstract

Antithrombotics require careful monitoring to prevent adverse events. Safe use can be promoted through so-called antithrombotic stewardship. Clinical decision support systems (CDSSs) can be used to monitor safe use of antithrombotics, supporting antithrombotic stewardship efforts. Yet, previous research shows that despite these interventions, antithrombotics continue to cause harm. Insufficient adoption of antithrombotic stewardship and suboptimal use of CDSSs may provide and explanation. However, it is currently unknown to what extent hospitals adopted antithrombotic stewardship and utilize CDSSs to support safe use of antithrombotics. A semi-structured questionnaire-based survey was disseminated to 12 hospital pharmacists from different hospital types and regions in the Netherlands. The primary outcome was the degree of antithrombotic stewardship adoption, expressed as the number of tasks adopted per hospital and the degree of adoption per task. Secondary outcomes included characteristics of CDSS alerts used to monitor safe use of antithrombotics. All 12 hospital pharmacists completed the survey and report to have adopted antithrombotic stewardship in their hospital to a certain degree. The median adoption of tasks was two of five tasks (range 1–3). The tasks with the highest uptake were: drafting and maintenance of protocols (100%) and professional’s education (58%), while care transition optimization (25%), medication reviews (8%) and patient counseling (8%) had the lowest uptake. All hospitals used a CDSS to monitor safe use of antithrombotics, mainly via basic alerts and less frequently via advanced alerts. The most frequently employed alerts were: identification of patients using a direct oral anticoagulant (DOAC) or a vitamin K antagonist (VKA) with one or more other antithrombotics (n = 6) and patients using a VKA to evaluate correct use (n = 6), both reflecting basic CDSS. All participating hospitals adopted antithrombotic stewardship, but the adopted tasks vary. CDSS alerts used are mainly basic in their logic.

## Introduction

Antithrombotics are one of the medication classes most commonly involved in medication errors and adverse drug events (ADEs). Of all unplanned hospital admissions, 5.6% of the admissions are medication related and antithrombotics belong to the top five medications involved in potentially preventable hospital admissions related to medication (HARMs) [1, 2]. In the inpatient setting, antithrombotics are also the drugs most frequently involved in ADEs and fatal ADEs [3]. In the Netherlands, as a response to the significant patient harm due to antithrombotics, several recommendations for their safe use were published: in 2008 in the HARM Wrestling report [4], and in 2012 in the guideline on the integrated antithrombotic care (Landelijke Standaard Ketenzorg Antistolling, LSKA in Dutch) [5]. A second version of LSKA was issued in 2014, due to the introduction of the direct oral anticoagulants (DOACs) and the resulting changes in the organization of anticoagulation care (decreasing role of thrombosis services). Additionally, the second version addresses the use of antiplatelet agents.

The LSKA contained different recommendations on providing optimal care to patients on antithrombotic therapy, describing tasks and responsibilities of healthcare providers [5]. Efforts are needed in Dutch hospitals to be able to fully implement the LSKA guideline and to carry out the tasks needed to improve the effects and safety of antithrombotic therapy. This could be achieved by multidisciplinary antithrombotic teams, also called antithrombotic stewardship. Antithrombotic stewardship has been promoted in other countries as well [6–8]. A study of Dreijer et al. showed the effect of antithrombotic stewardship: a reduction in bleeding episodes and thrombotic events from hospitalization until three months after hospitalization (14.3% during usual care versus 13.1% in intervention period) [9]. In another study it was found that introduction of antithrombotic stewardship led to a higher overall adherence to antithrombotic guidelines among prescribing physicians (adjusted odds ratio 1.58, 95% confidence interval 1.21–2.05) [10].

Optimization of clinical decision support systems (CDSSs) used by physicians and pharmacists was one of the recommendations of the Dutch HARM-wrestling Taskforce [4]. A CDSS automatically generates medication safety alerts by rule-based algorithms linking the content of a knowledge database with the content of the electronic health record (EHR) [11]. Besides the automatically generated alerts based on the knowledge database, hospitals can also build rules themselves that will trigger alerts or electronic reports for identifying risk patients. Various studies have demonstrated the potential of CDSSs to monitor safe use of antithrombotics and thereby reducing ADEs, for example in identifying patients on combined antithrombotic therapy [12–16].

Despite the recommendations of the LSKA and the HARM-Wrestling Taskforce, antithrombotics continue to substantially contribute to patient harm in hospitals with 16% of all preventable ADEs being related to antithrombotics in 2015/2016 and 21% in 2019 in The Netherlands [17, 18]. Insufficient implementation of the recommendations may explain these findings. Yet, since the publication of LSKA 2.0, only one study was performed on the implementation of the LSKA 2.0 in Dutch hospitals, but this mainly focused on the implementation and structure of network care [19]. Internationally, research on the adoption of antithrombotic stewardship is also scarce and the tasks studied are of limited scope or differ largely from the tasks specified in LSKA [7, 20–23]. In addition, the extent to which CDSSs are used in hospitals to support monitoring safe use of antithrombotics is unknown. Insights in antithrombotic stewardship adoption and utilization of CDSS to monitor safe use of antithrombotics are necessary to optimize medication safety around this high-risk drug class.

Therefore, the primary aim of this study is to evaluate the degree of adoption of antithrombotic stewardship tasks. The secondary aim is to describe the characteristics of CDSS used in the hospitals for medication surveillance of antithrombotics, to support antithrombotic stewardship efforts.

## Materials and methods

The survey was reported using the Consensus-Based Checklist for Reporting of Survey Studies (CROSS) [24]. The checklist can be found in S1 File.

### Study design

A cross-sectional study was performed, using a structured questionnaire-based survey. The questions included in the survey were formulated using the Systems Engineering Initiative for Patient Safety (SEIPS) 2.0 framework. This framework consists of five key components: people, tasks, tools and technology, environment, and organization [25, 26]. Using these five key components, the following topics were surveyed; information about the roles and responsibilities of healthcare professionals involved in antithrombotic stewardship was gathered (people), the responsibilities of the antithrombotic stewardship were explored (tasks), the CDSS systems utilized to monitor safe use of antithrombotics (tools and technology) and questions focused on environmental and organizational aspects of the antithrombotic stewardship. The survey can be found in S2 File.

No patient data were collected with this survey, so informed patient consent was not applicable. The respondents provided written informed consent by participating in the survey. Furthermore, the study did not fall under the scope of the Medical Research Involving Human Subjects Act. A waiver was obtained from the Medical Ethics Review Board of the University Medical Center Groningen (METC nr. 2023/477). The participants were asked to fill in their affiliations, but were not named in this paper to provide anonymity and confidentiality.

### Setting

In the Netherlands, case management concerning Vitamin K Antagonist (VKAs) for outpatients, is predominantly handled by specialized thrombosis services. This care entails regular international normalized ratio (INR) measurements to guide the adjustment of VKA dosages within a specified target range. The introduction of the Direct Oral Anticoagulants (DOACs) has led to a reduction in demand for thrombosis services in the Netherlands. Within some hospitals, thrombosis services play a role in dosing VKA, but in most hospitals this is performed by doctors or specialized nurses.

All Dutch hospitals use CDSSs in which medication safety alerts are triggered based on rules of the ‘G-standaard. The G-standaard is a national drug database maintained by the Royal Dutch Association for the Advancement of Pharmacy (KNMP in Dutch). The G-standaard contains logic-rules for inappropriate drug dosages, drug-drug interactions, contra-indications, duplicate medication, drug allergies and intolerances. These rules are decision-tree like rules consisting of multiple steps of ‘if then else’ logic to generate medication safety alerts based on data in the EHR. In addition, hospitals can develop logic-rules in-house to trigger medication safety alerts or electronic reports for patients at risk for ADEs. The logic used can vary from basic (combining one or two parameters to provide alerts, e.g. IF drug A AND drug B THEN alert), to a more advanced one (combining multiple patient characteristics when producing alerts, e.g. IF patient has characteristic X AND Y AND Z AND uses drug A AND has laboratory value > specified threshold THEN alert) [11].

### Study population

This study included a convenience sample of 12 Dutch hospital pharmacists from 12 different hospitals, representing 17.4% of the total number of Dutch hospitals (12/69) [27]. These 12 Dutch hospital pharmacists were selected since they participated in a project concerning use of artificial intelligence (AI) in optimizing CDSS alerts. At the time this network of 12 hospital pharmacists was established, the focus on antithrombotics and/or stewardship had not yet been defined. The sample comprised of two university hospitals, seven non-university teaching hospitals and three general hospitals. Geographically, four of these hospitals were situated in the northern region of the Netherlands, five in the central region and three in the southern region. The hospital pharmacists who completed the questionnaire were either actively involved in the antithrombotic stewardship themselves or consulted those within the hospital pharmacy as needed for questionnaire completion. The participating hospital pharmacists provided responses pertaining to overall hospital practices concerning antithrombotic stewardship, not only pharmacy-related practices. No methods were applied to adjust for possible non-representativeness of the sample.

### Survey administration

The survey was presented in Dutch and was conducted online using Research Electronic Data Capture (RedCap), version 12.4.6 (Vanderbilt University, Nashville, United States) as a data collection tool. Participants were asked to complete the questionnaire via email. These emails were sent on 25^th^ of May 2023 and the recruitment period ended on the 19^th^ of September 2023. All hospital pharmacists received a unique link to the questionnaire, to prevent unauthorized access to the questionnaire. Multiple participation via the unique link was prevented by asking to fill in the affiliations. When any uncertainty in the data or missing data were found, the participating hospital pharmacist was contacted by the research team to elucidate. When no responses were received, a reminder was sent after two weeks which was repeated after two more weeks when needed.

### Data collection

A combination of open ended and closed questions was used in the survey. The survey consisted of three parts. The first part was about the hospital characteristics, namely the hospital type, the number of beds and which EHR system is used. The second part was about the presence of antithrombotic stewardship and its tasks. The tasks as mentioned in the study of Dreijer et al. (a study exploring the effect of antithrombotic stewardship on thrombotic events and bleedings [9]) were assessed: drafting and maintenance of protocols, professional’s education, care transition optimization, medication reviews and patient counseling. Care transition optimization involved enhancing the efficiency and safety of processes related to a patient’s admission and discharge, such as providing structured pharmacotherapy advices to the thrombosis service (in case of VKAs), the general practitioner and the community pharmacist at discharge. A medication review consisted of checking dosages (e.g. in relation to renal function, bodyweight, age and indication), duplicate medication, drug-drug interactions, contraindications, perioperative bridging of antithrombotic treatment during surgery or interventions and over/-or undertreatment. Patient counselling aimed to provide information and education to patients, in addition to the information that is given by the pharmacy at discharge. All these tasks were specifically focusing on antithrombotics. The third part was about the characteristics of the CDSSs to monitor safe use of antithrombotics. It was examined which rules and/or reports are used on top of the standard rules included in the ‘G-standaard’. The participants were asked for a description of these rules. In addition, questions were included on which ‘G-standaard’ alerts concerning antithrombotics were suppressed, and on registration of ADEs due to antithrombotics.

Three hospital pharmacists were asked to pilot the initial survey. These three hospital pharmacists were all experts in the field of antithrombotic stewardship and were not part of the convenience sample of 12 hospital pharmacists. Adjustments were made based on the recommendations of these experts.

### Primary and secondary outcomes

The primary outcome was the level of antithrombotic stewardship adoption expressed as the proportion of antithrombotic stewardship tasks that were adopted per hospital [28]. Secondary outcomes were the characteristics of CDSSs used in the hospitals to support the tasks of antithrombotic stewardship.

### Data analysis

The data from the survey collected with RedCap were transferred to Excel version 2307 (Microsoft, Redmond, Washington, United States). Descriptive statistics were used to analyze the data. Continuous variables were expressed as mean with standard deviation (SD) if normally distributed or as median with range (minimum through maximum value) for non-normally distributed variables. Normality was checked using the Shapiro-Wilk test. Categorical variables were expressed as numbers and percentages.

## Results

### Respondents characteristics

The survey was completed by 12 hospital pharmacists, resulting in a response rate of 100%. The variation in the type of hospital in our sample differs from the hospital landscape in The Netherlands, with more university and less general hospitals, but resembles the geographic spread (Table 1). Seven hospitals used Hix^®^ (Chipsoft, Amsterdam, The Netherlands) and five hospitals used Epic^®^ (Verona, United States) as EHR system. All underlying data can be found in S1 Dataset.

**Table 1:** Respondent characteristics.

Characteristic	Number of participants (%)
Region of the Netherlands *	
• North	4 (33)
• Middle	5 (42)
• South	3 (25)
Type of hospital *	
• University	2 (17)
• Teaching	7 (58)
• General	3 (25)
EHR system used	
• Hix^®^	7 (58)
• Epic^®^	5 (42)
	Mean (SD)
Number of beds	
• University	1162 (195)
• Teaching	746 (176)
• General	317 (76)

*In the Netherlands, there are 7 (10%) university hospitals, 26 (38%) teaching hospitals, and 36 (52%) general hospitals. These hospitals are located in the North (12, 20%), the Middle (30, 43%), and the South of the Netherlands (25, 36%).

### Level of antithrombotic stewardship adoption

All hospitals mentioned having an antithrombotic stewardship team. The median proportion of adopted tasks per hospital was two tasks (range 1–3) (40% of tasks). None of the hospitals adopted all tasks included in the antithrombotic stewardship (Table 2).

**Table 2:** Adopted tasks of the antithrombotic stewardship per hospital.

	Drafting & maintaining protocols	Professional’s education	Care transition optimization	Medication reviews	Patient counseling	Total
Teaching hospitals						
1	X	X		X		3 (60%)
4	X	X				2 (40%)
5	X					1 (20%)
6	X		X			2 (40%)
8	X	X			X	3 (60%)
9	X	X				2 (40%)
11	X					1 (20%)
Median (range)						2.0 (1–3)
General hospitals						
2	X					1 (20%)
3	X					1 (20%)
10	X	X	X			3 (60%)
Median (range)						1.0 (1–3)
University hospitals						
7	X	X				2 (40%)
12	X	X	X			3 (60%)
Median (range)						2.5 (2–3)
Total	12 (100%)	7 (58%)	3 (25%)	1 (8%)	1 (8%)	

The tasks with the highest uptake were drafting and maintenance of protocols (100%), professional’s education (58%). The tasks with the lowest uptake were care transition optimization (25%), medication reviews (8%) and patient counseling (8%) (Table 2). Other tasks and characteristics of the tasks of the antithrombotic stewardship are described in Table 3.

**Table 3:** Characteristics of the stewardship and stewardship tasks and description of (additional) performed tasks of the antithrombotic stewardship.

Characteristics of the stewardship and tasks or description of tasks	Number of participants (%)
Additional tasks carried out as part of the antithrombotic stewardship, besides the tasks mentioned in the study of Dreijer et al. (n,%)	Total: 12
• Measurement and/or analysis of ADEs related to antithrombotics*	8 (67)
• Consulting on complex patients*	7 (58)
• Improving the EHR with respect to antithrombotic incidents, such as building clinical rules or improving the prescription system**	2 (17)
• Regional coordination of antithrombotic policy*	1 (8)
Main members of the antithrombotic stewardship (n≥5) (n,%)	Total: 12
• Hospital pharmacist	12 (100)
• Internist	12 (100)
• Surgeon	12 (100)
• Cardiologist	10 (83)
• Anesthesiologist	10 (83)
• Hematologist	9 (75)
• Clinical chemist	8 (67)
• Neurologist	8 (67)
• Clinical pharmacologist	5 (42)
• Pulmonologist	5 (42)
• Orthopaedic surgeon	5 (42)
Healthcare professionals that are provided education (n, %)	Total: 7
• Residents	7 (100)
• Medical specialist	6 (86)
• Nurse practitioners	6 (86)
• Nurses	4 (57)
• Physician assistants	4 (57)
• Pharmacy technicians	1 (14)
Frequency that healthcare professionals are provided education (n, %)	Total: 7
• No fixed frequency	2 (29)
• Unknown	2 (29)
• All new prescribers and yearly	1 (14)
• 1 time per year	1 (14)
• 2–3 times per year	1 (14)
Form of education (n,%)	Total: 7
• E-learning & lecture	3 (43)
• Lecture	1 (14)
• Written teaching materials & lecture	1 (14)
• Digital pocket card & lecture	1 (14)
Patients that receive medication reviews	Total: 1
• Patients suggested by a medical specialist or the pharmacist on duty and risk patients defined as all patients using a VKA or three or more antithrombotics	1 (100)

* This task is also mentioned in the LSKA

** This task is also mentioned in the HARM-Wrestling report

One hospital pharmacist mentioned having a physician who is fulltime working for the antithrombotic stewardship and can be consulted for patient cases. Another hospital pharmacist mentioned that a working group for patient safety regarding antithrombotics was established and a case manager assigned. The naming varied between the hospitals, but three other hospital pharmacists mentioned having an antithrombotic committee and antithrombotic stewardship (in two of the cases the stewardship is in the process of being formed). In these hospitals the committee is mainly responsible for drafting and maintaining protocols and antithrombotic policy, while the antithrombotic stewardship handles consultation on complex patients.

The one hospital which conducts medication reviews, emphasizes a focus on anticoagulants during these assessments. Additionally, the hospital providing patient counselling offers this service to all patients at the commencement of anticoagulation therapy, with periodic informational sessions for patients.

### Secondary outcomes

Regarding utilization of CDSS, 11 hospitals (92%) used clinical rules, best practice alerts and/or electronic reports in addition to standard alerts generated via the ‘G-standard’ (Table 4). The first four rules shown in Table 4 reflect basic CDSS alerts, since only two or three patient characteristics are taken into account, e.g. medication use and lab results. Identifying patients in need of thromboprophylaxis or all high-risk patients concerning antithrombotics requires inclusion of at least five patient characteristics, reflecting advanced CDSS.

**Table 4:** Respondent descriptions of additional alerts to monitor safe use of antithrombotics.

Description of additional alerts	Number of participants (%)
Identification of patients using a DOAC or a VKA in combination with one or more other antithrombotics to be able to check the indications	6 (50)
Identification of patients using a VKA to see whether information was noted for the thrombosis service at discharge, whether an INR was established daily and/or whether this INR was within therapeutic range	6 (50)
Identification of all patients using a DOAC to evaluate whether the kidney function, weight and/or age was established and matched the current dose	4 (33)
Identification of patients using VKA together with an LMWH having an INR within therapeutic range	3 (25)
Identification of all patients in need of thromboprophylaxis (in combination with the Padua prediction score [29])	3 (25)
Identification of all high risk patients concerning antithrombotics; patients in need of an individual dosage based on characteristics such as weight, age, kidney function and CYP2C19 metabolism	1 (8)

DOAC = direct oral anticoagulant, INR = international normalized ratio, LMWH = low molecular weight heparin, VKA = vitamin K antagonist

In five hospitals (42%) some of the ‘G-standard’ alerts concerning antithrombotics were suppressed. These were alerts related to interactions between two antithrombotics or interactions between VKA’s and other medication causing a change in INR. These alerts were suppressed mainly because they were replaced by more advance rules e.g. combining dosing with INR or kidney function.

## Discussion

All hospitals adopted antithrombotic stewardship but none reached full adoption of all tasks. The median adoption percentage of the antithrombotic stewardship tasks was 40%. Tasks with the highest uptake were drafting and maintenance of protocols (100%) and professional’s education (58%). In addition to G-standard alerts, 92% of hospitals use additional rules or reports, however these are mainly basic alerts since they combine only a few patient characteristics. Only four hospitals use more advanced CDSS alerts: one hospital identifies all high-risk patients concerning antithrombotics taking age, weight, renal function and genetics into account and three other hospitals use a tool in the EHR to calculate Padua prediction scores to identify non-surgical patients potentially needing thromboprophylaxis.

In the Netherlands, the timeline concerning the implementation of antithrombotic stewardships consists of the following most important milestones; the HARM-study was published in 2008, which lead to the publication of the LSKA guideline in 2012 (vs 1, vs 2 in 2014) and the study of Dreijer et al. on the effect of antithrombotic stewardship on the efficacy and safety of antithrombotic therapy was published in 2020. Research findings, such as the HARM-study, are only the starting point for improving healthcare in clinical practice. The effort it takes to translate research findings into valid and effective clinical practice has been widely underestimated [30]. Different studies have shown that on average it takes about 17 years for research findings to reach clinical practice [31–35]. This time lag can also be observed in the implementation of antithrombotic stewardship as shown by our findings.

The absence of practical implementation recommendations may explain this time lag. No practical guidelines were stated how to implement antithrombotic stewardship, contrary to the implementation of an antimicrobial stewardship. For example, the Dutch Foundation Working Group Antibiotics Policy (In Dutch: Stichting Werkgroep Antibiotica Beleid (SWAB)) published a practical guide as a tool for establishing and implementing antimicrobial stewardship [36]. Furthermore, Mendelson et al. described how to start an antimicrobial stewardship program in their hospital, the components of such a program and the evidence base for its implementation [37]. No practical guidelines were stated how to implement the tasks described by the study of Dreijer et al., contrary to the implementation of an antimicrobial stewardship. This emphasizes the need to study the factors that influence implementation of the antithrombotic stewardship [38].

Other reasons why the antithrombotic stewardship is not fully implemented may be lack of financial resources, lack of key personnel, competition from other priority initiatives or just simply aiming at other goals with the stewardship then described by the study of Dreijer et al. Similar barriers were faced when implementing antimicrobial stewardship in hospitals [39].

The logic of CDSS rules used in the participating hospitals was relatively basic taking into account just a few patient characteristics. The fewer characteristics are taken into account, the less patient specific the alerts are, consequently leading to a higher amount of non-relevant alerts and alert fatigue [11]. Therefore, more advanced CDSS is needed to optimize safe us of antithrombotics [11, 40, 41]. Recently use of AI has been proposed as the next frontier in improving effectivity of CDSSs by improving and/or developing personalized medication safety alerts [42–45]. Using AI, advanced algorithms could be developed to prevent ADEs due to the use of antithrombotics.

To the best of our knowledge, this is the first study on the adoption of antithrombotic stewardship and also the first study on the utilization of CDSSs to monitor safe use of antithrombotics. A strength of this study is that we used the SEIPS framework to construct our survey and by doing so investigating a broad range of factors that may influence adoption of antithrombotic stewardship. Furthermore, all questionnaires were returned reducing the risk of response bias.

A limitation of this study is that we used a convenience sample consisting of 17% of all Dutch hospitals, which may limit generalizability and introduce selection bias. Although our sample differs from the hospital landscape in The Netherlands, all types of hospitals were represented as well as all regions of the Netherlands to enhance generalizability and limit selection bias. The 12 participants were selected based on their participation in a project concerning the use of artificial intelligence in optimizing CDSS alerts, which limits selection bias. On the other hand, given their interest in optimizing CDSS it may be argued that they are more likely to adopt CDSS and antithrombotic stewardship. Therefore the results may even be overestimated. Another limitation is that a survey was used in this study, which may lead to the fact that questions were interpreted differently among the participants and an in-depth analysis of the reasons for incomplete adoption was not possible. Additional studies using interviews or focus groups are needed to resolve these considerations. Finally, hospital pharmacists may not be aware of activities related to antithrombotic stewardship of other healthcare professionals in the hospital, leading to underreporting. However, in the Netherlands hospital pharmacists are in general widely involved in all hospital care regarding antithrombotics, making this limitation less likely.

In future research it is necessary to obtain further insights into the barriers and facilitators healthcare professionals experience with antithrombotic stewardship implementation. Practical issues should be addressed, but also lessons learned from already implemented tasks of the antithrombotic stewardship. In addition, the potential of AI to develop more personalized antithrombotic alerts should be further explored, to support healthcare professionals in antithrombotic stewardship tasks.

## Conclusion

Findings of this study reveal that all participating hospitals adopted an antithrombotic stewardship. However, tasks of antithrombotic stewardship are only partly adopted and vary across hospitals. Furthermore, CDSS alerts used to monitor safe use of antithrombotics are mainly basic in their logic, with just a few more advanced alerts implemented. To optimize safe use of antithrombotics, all tasks need to be adopted completely and broader use of more advanced CDSS may facilitate this.

## Supporting information

S1 File Consensus-Based checklist for reporting of survey studies (CROSS). (DOCX)

S2 File Survey. (DOCX)

S1 Dataset (XLSX)
