# Supplementary material for: Adoption of antithrombotic stewardship and utilization of clinical decision support systems—A questionnaire-based survey in Dutch hospitals
Source: PLoS One. 2024 Jun 21;19(6):e0306033. doi: 10.1371/journal.pone.0306033 (PMC11192363; doi:10.1371/journal.pone.0306033)
Supplement: S2 File — (DOCX) [file pone.0306033.s002.docx]

1. What is the name and the location of the hospital
2. What type is the hospital?
   - General
   - Teaching
   - Universitary
3. How many beds does the hospital have?
4. What hospital information system is used in the hospital?
   - Hix/Chipsoft
   - Epic
   - Nexus
   - Otherwise, namely:
5. Is there an antithrombotic team/-committee and/or pharmacist who focuses on antithrombotics? (multiple answers possible)
   - Antithrombotic team/-committee
   - Pharmacist with a focus on antithrombotics
   - No
   - Otherwise, namely:
6. If an antithrombotic team/-committee is present; Who are part of this team/-committee? (multiple answers possible)
   - Nurse
   - Internist
   - Hospital pharmacist
   - Clinical pharmacologist
   - Cardiologist
   - Anesthesiologist
   - Pulmonologist
   - Pediatrician
   - Neurologist
   - Surgeon
   - Emergency physician
   - Clinical chemist
   - Hematologist
   - Otherwise, namely:
7. If an antithrombotic team/-committee is present; What tasks does this team, this committee or this specialized pharmacist perform? (multiple answers possible)
   - Professional’s education
   - Medication reviews
   - Consultation on complex patients
   - Drafting and maintenance of protocols
   - Patient counseling
   - Care transition optimization
   - None of the above
   - Otherwise, namely:
8. If professional’s education is performed; How often is education about antithrombotics provided?
9. If professional’s education is performed; In what form/way is education about antithrombotics provided?
10. If professional’s education is performed; For which healthcare professionals is education on antithrombotics offered (multiple answers possible)
    - Medical specialists
    - Residents
    - Nurses
    - Nurse practitioners
    - Physician assistants
    - Otherwise, namely:
11. If medication reviews are performed; For which patient categories are medication reviews performed? (multiple answers possible)
    - Children
    - Elderly
    - Risk patients, namely:
    - Otherwise, namely:
12. If medication reviews are performed; Are these medication reviews specifically aimed at antithrombotics and/or is there explicit attention to antithrombotics?
    - Yes, namely:
    - No, because:
13. If patient counseling is provided; To which patient category/categories is information provided about antithrombotics? (multiple answers possible)
    - All patients
    - Children
    - Elderly
    - Risk patients, namely:
    - Otherwise, namely:
14. What type of CDSS alerts are utilized to monitor safe use of antithrombotics? (multiple answers possible)
    - ‘G-standaard’ alerts
    - Clinical rules
    - Best practice alerts
    - Electronic reports
    - Otherwise, namely:
15. If clinical rules are used; Provie a brief description of the clinical rules that are utilized pertaining to antithrombotics.
16. If best practice alerts are used; Provide a brief description of the best practice alerts utilized pertaining to antithrombotics.
17. If electronic reports are used; Provide a brief description of the electronic reports utilized pertaining to antithrombotics.
18. If other forms of medication surveillance with CDSS alerts are used; Proivde a description of the other methods pertaining to antithrombotics.
19. Are measurements and/or analyses carried out about adverse drug events related to antithrombotics?
    - Yes, namely:
    - No
20. Are any G-standard alerts disabled that specifically relate to antithrombotics?
    - Yes, namely:
    - No
